# Development of an internalin-based double-antibody sandwich quantitative ELISA for the detection of *Listeria monocytogenes* in slaughterhouse environments

**DOI:** 10.3389/fvets.2025.1517845

**Published:** 2025-03-07

**Authors:** Qing Cao, Wenjing Shi, Yanquan Wei, Jiayu Wang, Zhonglong Wang, Qian Chong, Qianqian Guo, Kunzhong Zhang, Wenyan Gai, Huitian Gou, Huiwen Xue

**Affiliations:** ^1^College of Veterinary Medicine, Gansu Agricultural University, Lanzhou, China; ^2^Shandong Vocational Animal Science and Veterinary College, Weifang, China

**Keywords:** *Listeria monocytogenes*, internalin G, monoclonal antibodies, slaughterhouse, DAS-qELISA method

## Abstract

**Introduction:**

*Listeria monocytogenes* causes zoonotic listeriosis with a high mortality rate, which is frequently detected in slaughterhouse processing environments and animal-based food. To enable the specific, rapid, and cost-effective detection of *L. monocytogenes* in environments and animal-based food, we developed a double-antibody sandwich quantitative ELISA (DAS-qELISA) method.

**Methods:**

The method is based on monoclonal antibodies targeting internalin G (InlG), a surface protein of *L. monocytogenes* with demonstrated immunogenicity. The antibody pair 1D2-2H10 was selected for use in the sandwich ELISA format. Optimization of the DAS-qELISA method was carried out to determine its detection limits for InlG protein and *L. monocytogenes*.

**Results:**

The detection limits of the method were determined to be 32 ng/mg for the InlG protein and 7875.83 CFU/mL for *L. monocytogenes*. The accuracy of the method was evaluated across various bacterial concentrations, with results falling within 91.56–107.07% and a coefficient of variation (CV) of less than 10%. Compared to traditional methods, this approach requires only 12 h of bacterial enrichment and incubation to achieve 100% accuracy.

**Discussion:**

The DAS-qELISA developed in this study provides a rapid, accurate, and cost-effective tool for the detection of *L. monocytogenes* in environmental and animal-based food samples. This method could be a valuable addition to current diagnostic approaches, offering quicker turnaround times and high accuracy for pathogen detection.

## 1 Introduction

*Listeria monocytogenes*, a Gram-positive foodborne pathogen of significant notoriety, possesses an intrinsic ability to invade and persist across various biological barriers, including the intestinal, placental, and blood–brain barriers (1–3). This microorganism poses a profound risk to immunocompromised individuals, where it can precipitate severe clinical manifestations such as bacteremia, sepsis, and meningitis. Its exceptional adaptability, particularly its capacity to thrive under refrigerated conditions, renders *L. monocytogenes* a formidable contaminant in the meat and dairy industries, jeopardizing the safety of food processing, transportation, and storage. The escalating global incidence of *L. monocytogenes* infections has emerged as a critical concern within the public health sector (4). Between January 2017 and January 2018, South Africa encountered an unprecedented outbreak, with 820 cases of listeriosis reported, marking the largest single-country outbreak in history (5). In 2018, a multinational foodborne outbreak in Europe, attributed to *L. monocytogenes*, resulted in 32 confirmed cases and six fatalities (6). In China, the bacterium has been found with an average prevalence of 4.42% across 28 provinces, with all cases linked to foodborne sources (7). Studies have identified meat and aquatic products as the primary vectors of transmission, while the risk associated with ready-to-eat foods remains significant (8, 9). Consequently, the rapid and precise detection and control of *L. monocytogenes* within the food production chain is of crucial.

In the quest for efficient *L. monocytogenes* detection, a plethora of methodologies has been developed. Traditional detection approaches, adhering to the Chinese national standards for food safety, rely on culture isolation followed by Gram staining and biochemical identification, which are labor-intensive and time-consuming. The advent of molecular biology techniques, particularly PCR-based methods, has revolutionized the field by offering enhanced speed, sensitivity, and specificity. Real-time quantitative PCR (RT-qPCR), multiplex PCR, and digital PCR have become mainstays for rapid detection, reducing detection time and enabling the simultaneous identification of multiple pathogens, thereby improving the efficiency of food safety testing (10–12). However, these PCR methods necessitate specialized equipment, reagents, and skilled operators, and are prone to false positives, which limits their broader application in routine production. Immunological techniques, leveraging antigen–antibody specificity, have been extensively employed and continuously optimized for detecting *L. monocytogenes*. Techniques such as enzyme-linked immunosorbent assay (ELISA) (13), immunomagnetic separation (IMS) (14), and immunofluorescence (15) have significantly enhanced the sensitivity and specificity of Listeria detection. These approaches are user-friendly, cost-effective, and suitable for high-volume screening of various food and environmental sources for *L. monocytogenes*.

The cornerstone of immunological detection is the acquisition of pathogen-specific antibodies. Internalins, surface proteins on *L. monocytogenes* that facilitate host cell invasion, have been the subject of intensive research, with 25 internalins identified to date (16). Monoclonal antibodies (mAbs) have been developed against internalins A and B (17, 18). Among these, InlG, a high molecular weight protein with an LPXTG motif at its C-terminus, anchors it to the bacterial cell wall and is commonly found in environmental isolates and highly virulent strains (19). Our previous studies demonstrated the strong immunogenicity of InlG, as recombinant InlG subunit vaccines induced high antibody levels and conferred protective immunity in mice (20). Subsequent experiments utilized purified InlG protein to immunize BALB/c mice, employing hybridoma technology to produce specific mAbs. Three mAbs (1D2, 1D2-1, and 2H10) were successfully generated and each recognizing distinct epitope on the InlG protein (21). In this study, a pair of anti-InlG mAbs was selected, and a double-antibody sandwich quantitative ELISA (DAS-qELISA) was developed and optimized for the detection of *L. monocytogenes*. The assay exhibited high sensitivity, specificity, accuracy, and cost-effectiveness for the rapid detection of *L. monocytogenes* contamination in farm environments and carcass samples, highlighting its potential as a valuable tool in food safety diagnostics.

## 2 Materials and methods

### 2.1 Bacterial strains, cell lines, and culture media

Monoclonal antibodies directed against the InlG protein of *Listeria monocytogenes* ATCC 19111 were derived from the supernatants and ascites of hybridoma cell lines 1D2, 1D2-1, and 2H10. These mAbs, along with a polyclonal antibody (pAb) raised against *L. monocytogenes* ATCC 19111, have been prepared and maintained within our laboratory’s repository (21). The hybridoma cell lines 1D2, 1D2-1, and 2H10, which specifically target the InlG protein of *L. monocytogenes* ATCC 19111, were previously generated and conserved by our research group. The bacterial strains employed for experimental testing are listed in Table 1. Cultivation of *Listeria* spp., *Streptococcus agalactiae*, *Staphylococcus aureus*, and *Staphylococcus albus* was conducted overnight in Brain-Heart Infusion (BHI) medium at a temperature of 37°C. To ascertain the presence of *L. monocytogenes* in agricultural settings and on carcasses, samples were subjected to pre-enrichment using Luria-Bertani (LB) 1 and LB2 media, followed by incubation at 37°C. Similarly, *Escherichia coli* and *Salmonella* strains were cultured overnight in LB medium at the same temperature.

**Table 1:** Bacterial strains used in study.

Strains	Description	Source or reference
*Listeria monocytogenes ATCC19111*	*L. monocytogenes* Serotype 1/2a standard strain	Poultry
*Listeria monocytogenes ATCC19112*	*L. monocytogenes* Serotype 2 standard strain	Human
*Listeria monocytogenes ATCC19115*	*L. monocytogenes* Serotype 4b standard strain	Human
*Streptococcus agalactiae* ATCC13813	*S. agalactiae* standard strain typed as Lancefield’s group B	Cattle
*Staphylococcus aureus*	Isolated strain	Cattle
*Staphylococcus albus*	Isolated strain	Cattle
*Escherichia coli*	Isolated strain	Cattle
*Salmonella enteritidis*	Isolated strain	Cattle

### 2.2 Antibody potency and subclass detection

The efficacy of the antibodies was evaluated through ELISA. For the determination of antibody isotypes, the Pro-Detect Rapid Antibody Isotyping Assay Kit - Mouse (Thermo Scientific™, Shanghai) was utilized. In the process of evaluating antibody potency, the wells of the microtiter plate were coated with a solution containing 2 μg/mL of the InlG protein, which was then incubated at 4°C overnight. Following the coating, the plate was blocked with a 5% skimmed milk solution for 1 h at 37°C. The mAbs were diluted in a two-fold serial manner, introduced to the plate, and incubated at 37°C for 1 h. Post-incubation, the plate was subjected to a washing procedure. Subsequently, horseradish peroxidase (HRP) -conjugated goat anti-mouse IgG (Beyotime, Shanghai) was added as the secondary antibody and incubated at 37°C for 1 h. Thereafter, 3,3′,5,5′-tetramethylbenzidine (TMB) (Beyotime, Shanghai) was added to facilitate color development, which was allowed to proceed at room temperature in the dark for 15 min. The protocol provided by the manufacturer was followed for the identification of antibody subtypes. For the isotyping assay, immunoproteins at a concentration of 50 ng per well were used to coat the enzyme-linked plate. For each positive clone, 600 μL of supernatant was collected, and a 100 μL portion was transferred to six wells that had been coated with the relevant proteins. After an hour of incubation at 37°C, the plate was washed three times with phosphate buffered saline with tween 20 (PBST). Following this, secondary antibodies specific to IgM, IgA, IgG1, IgG2a, IgG2b, IgG3, *λ*, and *κ* were added to the respective wells, incubated at 37°C for 1 h, and then washed three times with PBST before TMB was used to develop color.

### 2.3 The selection of paired antibodies

To determine the optimal combination of capture and detection antibodies for the development of a sandwich ELISA, three mAbs targeting the InlG antigen and a rabbit pAb specific to *Listeria monocytogenes* were conjugated with HRP via the sodium periodate oxidation method, utilizing the HRP conjugation kit (Sangon Biotech Co., Ltd., Shanghai). For the conjugation process, 1 mg of the antibody to be labeled was introduced into a dialysis bag with a molecular weight cut-off exceeding 8,000 Da. The dialysis bag was then sealed and immersed in 2 liters of conjugation buffer, and the mixture was dialyzed overnight at 4°C. Subsequent to dialysis, the contents of the dialysis bag were transferred into a 5 mL centrifuge tube, to which 100 μL of a reducing agent was added, followed by incubation at room temperature for a duration of 2 h.

The HRP-conjugated antibodies were employed as detection antibodies, while their non-conjugated counterparts served as capture antibodies. An array-based strategy was implemented to evaluate the various antibody pairs in a sandwich ELISA format. The concentration of both capture and detection antibodies was maintained at 2 μg/mL. The *Listeria monocytogenes* strain ATCC 19111 was used for testing at two bacterial concentrations: 10^4^ and 10^7^ colony-forming units per milliliter (CFU/mL), with phosphate-buffered saline (PBS) acting as the negative control. The assay outcomes were quantified as the ratio of positive to negative absorbance (P/N) at a wavelength of 450 nm optical density (OD) for each antibody pair. Antibody pairs yielding a P/N value of ≥20 were identified as suitable candidates for subsequent evaluation in the sandwich ELISA protocol.

### 2.4 Optimized DAS-qELISA method

To establish the optimal conditions for antibody capture in the ELISA, the capture antibody was diluted in a series ranging from 0.1 to 5 μg/mL. The encapsulation protocol was conducted under the following thermal regimens: incubation at 37°C for 1 h, 37°C for 2 h, room temperature (RT) for 1 h, RT for 2 h, or 4°C overnight. Subsequent to the encapsulation process, the plates were subjected to blocking with either 1, 2%, or 5% bovine serum albumin (BSA) or an equivalent concentration of skimmed milk. The blocking conditions entailed incubation at 37°C for 1 or 2 h, RT for 1 or 2 h, or 4°C overnight.

Following the blocking step, the antigen was introduced to the plates and incubated under the identical thermal conditions as the blocking step: 37°C for 1 or 2 h or RT for 1 or 2 h. The detection antibodies were also diluted in a series from 0.1 to 5 μg/mL and incubated with the antigen under the same thermal conditions as previously described.

Color development was initiated by the addition of TMB, with incubation durations of 5, 10, 15, or 20 min at RT. Following color development, the optical density (OD) at 450 nm was quantified. The reaction condition that yielded the highest ratio of positive to negative absorbance (P/N ratio) was identified as the optimal condition for the ELISA protocol.

### 2.5 Evaluation of optimized DAS-qELISA method

InlG protein was subjected to a two-fold serial dilution, ranging from 10 to 5,120 ng/mL, and employed as the proteinic antigen. Similarly, *Listeria monocytogenes* ATCC 19111 was diluted ten-fold across a concentration series from 10 to 10^9^ colony-forming units (CFU) to serve as the bacterial antigen. Negative controls were established using 2% bovine serum albumin (BSA) and phosphate-buffered saline (PBS). A cut-off value was established at 2.1 times the optical density at 450 nm (OD450) of the negative control, and the minimum antigen concentration exceeding this threshold was designated as the detection limit. A standard curve was constructed by plotting the OD450 values on the y-axis against the antigen concentrations on the x-axis. Subsequent to this graphical representation, linear regression analysis was performed to derive the standard curve equation. The OD450 value for any given antigen concentration was computed using this equation and compared with the empirically determined value. Statistical analyses were conducted to calculate the mean, standard deviation (SD), standard error of the mean (SEM), coefficient of variation (CV), and recovery rate. The recovery rate was computed as a percentage, using the formula: (detected value/theoretical value) × 100%. Utilizing the optimized DAS-qELISA method, the detection of various bacterial strains was undertaken, including *L. monocytogenes* ATCC 19112, *L. monocytogenes* ATCC 19115, *L. innocua*, *Streptococcus agalactiae*, *Staphylococcus aureus*, *Staphylococcus albicans*, *E. coli*, and *Salmonella* spp. To evaluate the specificity of the ELISA, *L. monocytogenes* ATCC 19111 was employed as the positive control, while PBS served as the negative control in the specificity evaluation.

### 2.6 The detection of *L. monocytogenes* in artificially inoculated beef and milk

Fresh beef and milk specimens were procured from local retail establishments. Beef specimens were sectioned into 3 g increments. One such increment was homogenized and subsequently cultured on Brain Heart Infusion (BHI) agar to ascertain the presence of bacterial contaminants. The residual beef samples were preserved at −20°C. Following confirmation of the absence of bacterial contamination, manual inoculation was executed. Suspensions of *Listeria monocytogenes* were prepared at a series of concentrations from 5 × 10^4^ to 5 × 10^7^ colony-forming units per milliliter (CFU/mL). A volume of 10 μL of the suspension was injected into the beef samples, which were then allowed to stand at ambient temperature for a duration of 15 min. Subsequently, the samples were transferred to homogenization tubes, to which 2 mL of BHI broth was added, followed by incubation at 37°C. At predetermined time intervals, 100 μL aliquots were extracted for analysis via the DAS-qELISA assay.

For the milk samples, a volume of 100 μL was plated on BHI agar to screen for bacterial contamination, with the remaining specimens stored at 4°C. Once the absence of contamination was verified, manual inoculation was conducted. *Listeria monocytogenes* was diluted to achieve a concentration spectrum of 5 × 10^4^ to 5 × 10^7^ CFU/mL, and 10 μL of this suspension was introduced into 2.99 mL of milk. After the addition of 2 mL of BHI broth, the inoculated milk samples were incubated at 37°C. At various time points, 100 μL of the suspension was sampled for analysis via the DAS-qELISA assay. Concurrently, 100 μL of each artificially contaminated beef and milk sample was plated on BHI agar following serial dilution to facilitate colony enumeration.

### 2.7 The detection of *L. monocytogenes* in abattoirs samples

In the course of the sampling procedure, sterile cotton-tipped applicators saturated with sterile normal saline were utilized to swab various anatomical regions of livestock carcasses within abattoirs. This included the dorsal and ventral aspects of the forelimbs, hindlimbs, spine, abdomen, and ten distinct lateral body segments (with each segment encompassing a minimum area of 5 cm^2^). An equivalent method was employed for the swabbing of various surfaces within the slaughterhouse facilities. Each swab was subsequently transferred into a sterile centrifuge tube containing 5 mL of sterile normal saline and preserved at a temperature of 4°C.

For the detection of *Listeria monocytogenes* in carcass and environmental samples procured from abattoirs, the national standard method was implemented. The samples were inoculated into 50 mL centrifuge tubes containing 35 mL of LB1 broth supplemented with bacteriostatic agents and subjected to incubation at 30°C with agitation at 180 rpm for a period of 22–26 h to facilitate pre-enrichment. Subsequently, a 100 μL aliquot of the pre-enrichment broth was transferred into centrifuge tubes with 6 mL of LB2 broth and further incubated at 30°C with agitation at 180 rpm for 24 h to enhance enrichment. Using aseptic technique, bacterial suspensions were streaked onto Polymyxin-acriflavine-LiCl-ceftazidime-aesculin-mannitol (PALCAM) agar and *L. monocytogenes*-specific chromogenic medium via the three-zone streak method. The culture plates were then incubated statically at 37°C for 24 h. Colonies exhibiting a characteristic sunken center with gray edges on PALCAM agar, and smooth, round, blue-green colonies on the *L. monocytogenes* chromogenic medium, were classified as presumptive positive for *L. monocytogenes*.

For the detection of *Listeria monocytogenes* in carcass and environmental samples from abattoirs utilizing the DAS-qELISA method, the samples were transferred into 50 mL centrifuge tubes containing 35 mL of Tryptose Hydrochloride Broth (THB) and subjected to incubation at 37°C with agitation at 180 rpm for a duration ranging from 6 to 72 h to facilitate pre-enrichment. At predetermined time intervals, a 100 μL aliquot of the enriched bacterial suspension was extracted for subsequent analysis via DAS-qELISA. In parallel, for the molecular identification of *L. monocytogenes* in the same sample types using polymerase chain reaction (PCR), the pre-enrichment protocol was identical to that employed for the DAS-qELISA. At various time points during the pre-enrichment phase, 1 mL of the enriched bacterial suspension was centrifuged at 12,000 × g for 10 min to separate the cells from the growth medium. The resulting cell pellet was resuspended in 180 μL of TE (Tris-EDTA) buffer. The resuspended cells were then subjected to boiling for 10 min to lyse the bacterial cells, followed by a second centrifugation step at 12,000 × g for 10 min to isolate the supernatant containing the DNA template for PCR amplification. PCR was conducted using primers specifically designed to target the *hlyA* gene (5′-GCAGTTGCAAGCGCTTGGAGTGAA-3′ and 5′-GCAACGTATCCTCCAGAGTGATCG-3′) (22) and *prfA* genes (5′-AACCAATGGGATCCACAAG-3′ and 5′-ATTCTGCTAACAGC TGAGC-3′) (23) of *L. monocytogenes*.

### 2.8 Statistical analyses

Data were presented as the mean ± standard deviations (SD). GraphPad Prism version 8.0.1 was used for the statistical analysis, linear regression analysis and graph preparation.

## 3 Results

### 3.1 Potency and subtypes of mAbs

The efficacy and isotypes of the mAbs present in the culture supernatants were evaluated via indirect ELISA, and the findings are detailed in Table 2. The ELISA analysis indicated that the 1D2 mAb demonstrated a high potency, achieving a dilution factor of 1:4,000. In comparison, the remaining two mAbs exhibited potencies at a dilution factor of 1:2,000. Additionally, the isotype analysis of the mAbs in the culture supernatants from three cell lines revealed uniformity, with each mAb possessing IgG_1_ heavy chains and *κ* light chains. Notably, the mAbs derived from ascites showed a marked enhancement in potency, with the 1D2 mAb reaching a titer of 1:32,000, whereas the other two mAbs had potencies of 1:16,000, as documented in Table 2.

**Table 2:** Potency and subtype of mAbs.

Strains	supernatant antibody potency	Ascites antibody potency	heavy chain subtype	light chain subtype
1D2	1:4,000	1:32,000	IgG_1_	κ
1D2-1	1:2,000	1:16,000	IgG_1_	κ
2H10	1:2,000	1:16,000	IgG_1_	κ

### 3.2 Antibody pairing assay

**Figure 1:**
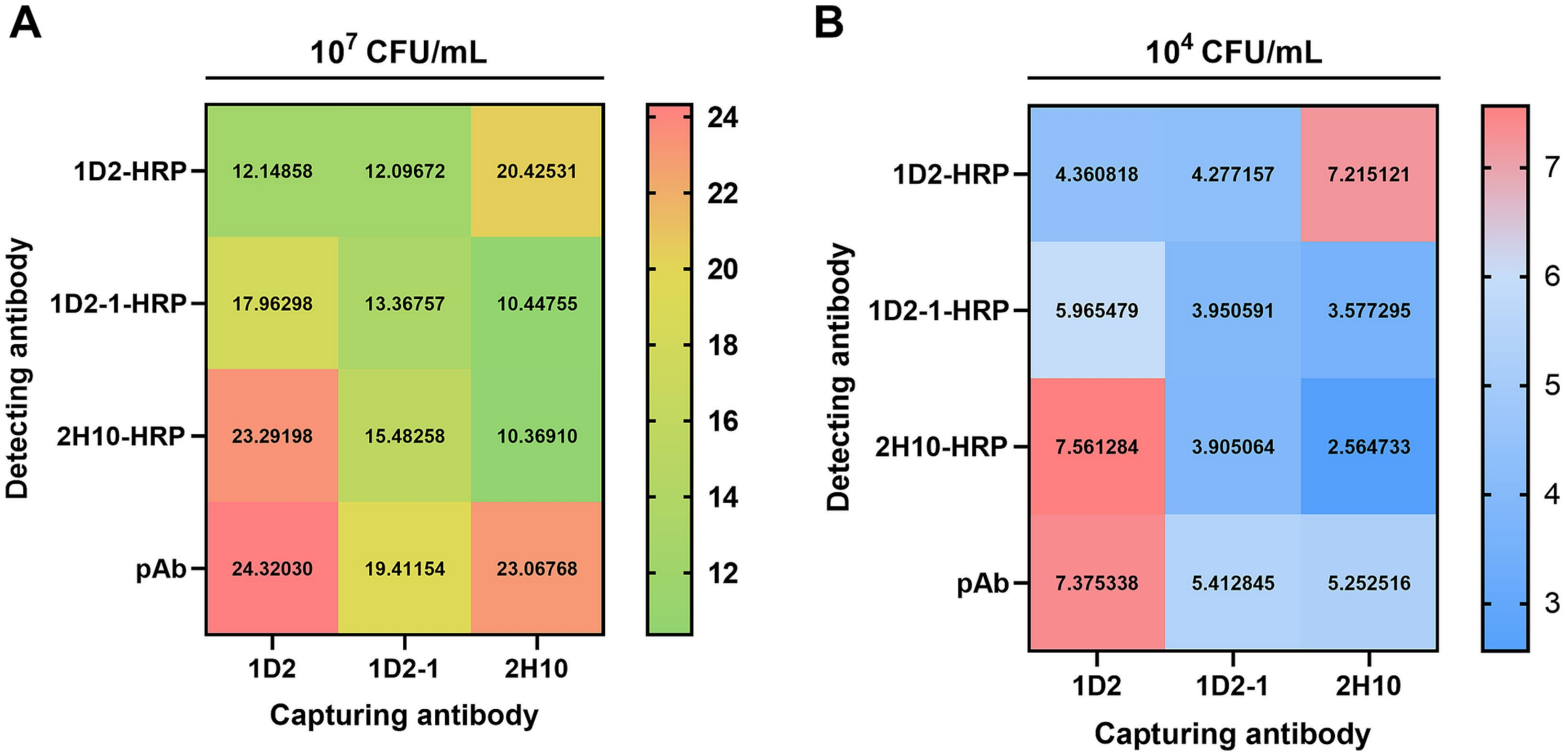
Sandwich ELISA checkerboard assay of capture and detection antibody pair against *L. monocytogenes*. 2 μg/mL of three capture Ab (1D2, 1D2-1, and 2H10) and 2 μg/mL of four detection Ab (1D2-HRP, 1D2-1-HRP, 2H10-HRP and pAb-HRP) were tested in various combinations against 10^7^ CFU/mL **(A)** and 10^4^ CFU/mL **(B)** *L. monocytogenes*.

In the development of an ELISA for the detection of InlG protein, three mAbs were evaluated in a paired configuration, with one serving as the capture antibody and the other as the detection antibody. Additionally, a pAb directed against *Listeria monocytogenes* was utilized as a control for the detection antibody. The results of the antibody pairing experiment revealed that when mAb 1D2 was employed as the capture antibody in conjunction with mAb 2H10-HPR as the detection antibody, the assay yielded a positive-to-negative (P/N) ratio exceeding 23 at high antigen concentrations (10^7^ CFU/mL) and 7.5 at low antigen concentrations (10^4^ CFU/mL). These P/N values were analogous to those achieved when mAb 1D2 was paired with the pAb across both high and low antigen concentrations, as depicted in Figure 1. Consequently, the 1D2:2H10-HPR antibody pairing was selected for subsequent investigations due to its favorable performance characteristics.

### 3.3 Evaluation of optimized conditions and assay capacity of DAS-qELISA

**Figure 2:**
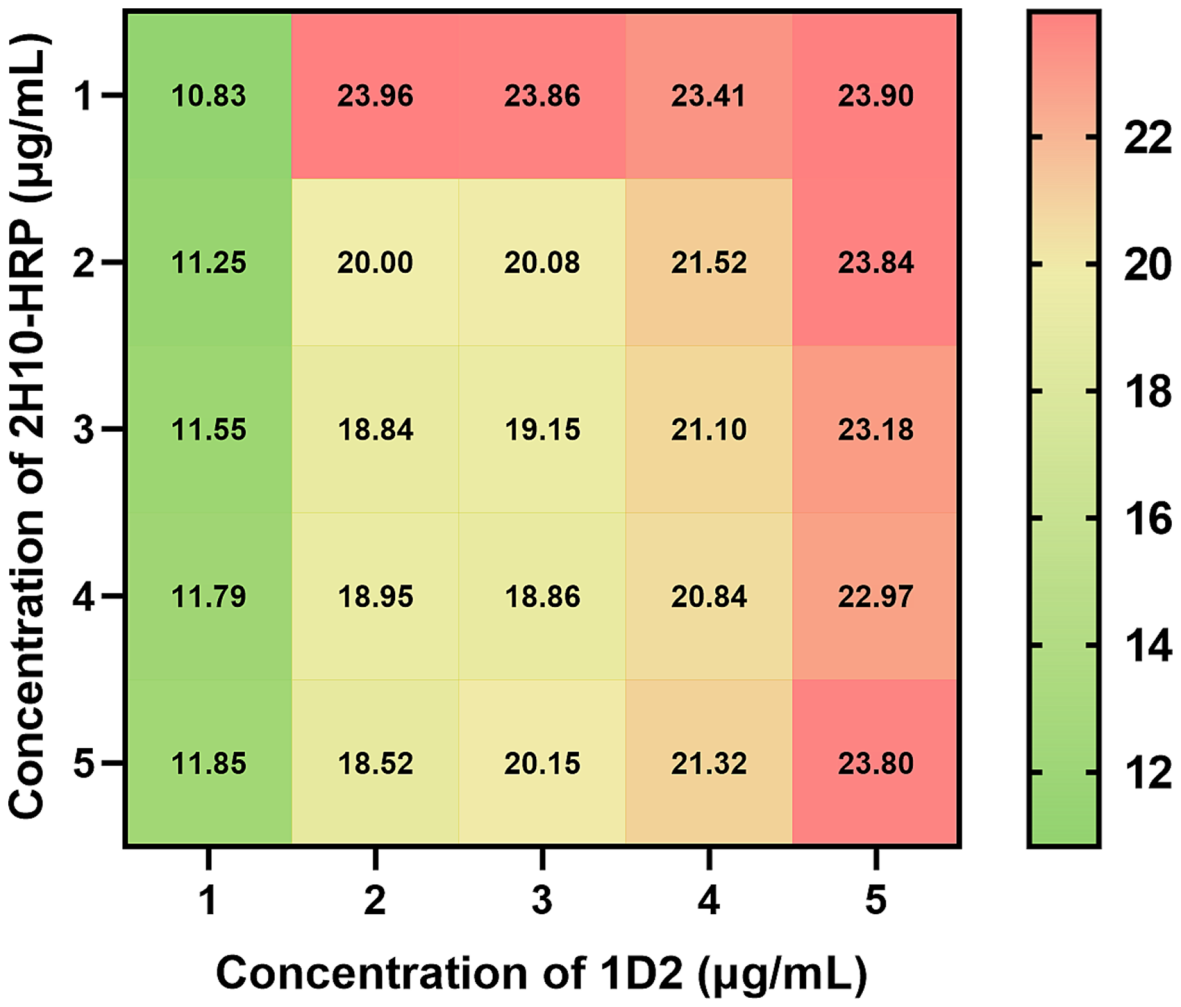
Checkerboard assay of optimized capture and detection antibody concentration against *L. monocytogenes*. A range of capture antibody 1D2 concentrations from 1 to 5 μg/mL, were incubated at 4°C for 16 h, followed by blocking with 2% BSA for 1 h at room temperature. Subsequently, 10^7^ CFU/mL *L. monocytogenes* were incubated for 2 h at room temperature. A range of detection antibody 2H10-HRP concentrations from 1 to 5 μg/mL, were incubated at room temperature for 2 h. The value in cells represent the P/N ratio of OD_450_ values to OD_450_ values of negative control.

The optimization of parameters for the sandwich ELISA, including the optimal concentrations of the antibody pair, the nature and duration of the blocking agent, the antigen incubation period, the detection antibody incubation duration, and the substrate reaction interval, was conducted utilizing a sandwich ELISA. The optimization of antibody pairing concentrations revealed that the maximum signal-to-noise ratio (P/N ratio) was attained with a capture antibody concentration of 1 μg/mL and a detection antibody concentration of 2 μg/mL (Figure 2). The refined experimental conditions entailed incubating the capture antibody at a concentration of 1 μg/mL for a duration of 16 h at 4°C, followed by blocking with 2% bovine serum albumin (BSA) for 1 h at ambient temperature. Subsequently, the antigen samples were incubated for 2 h at room temperature, the detection antibody was added at a concentration of 2 μg/mL and incubated for 1 h at room temperature, and finally, the substrate reaction was allowed to proceed for 15 min at ambient temperature.

The sensitivity of the developed ELISA assay was assayed using a range of concentrations of InlG protein and *L. monocytogenes* ATCC19111 as the target antigens. Negative controls for bacterial and protein detection were established using PBS and 2% BSA, respectively. A sample was deemed positive if the absorbance at 450 nm (OD_450_) exceeded 2.1 times the mean OD_450_ value of the respective negative control. The minimum detectable concentrations for InlG protein and *L. monocytogenes* ATCC19111 were defined as the detection limits.

**Figure 3:**
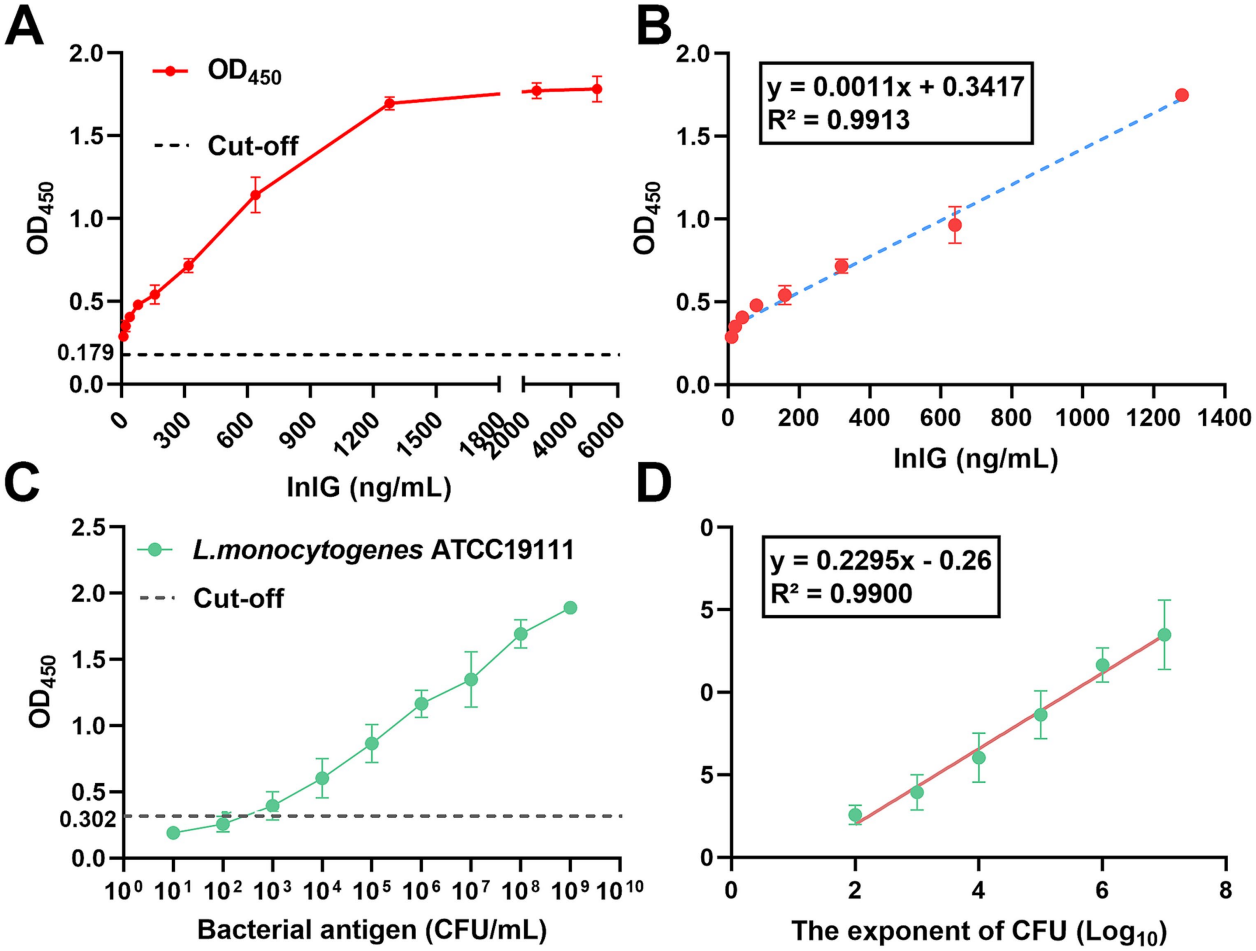
Detection limit and standard curve of DAS-qELISA based on 1D2 mAb and 2H10-HRP mAb for detecting InlG and *L. monocytogenes*. **(A)** The linear curve of the DAS-qELISA assay for the quantitative detection of serially diluted InlG protein 10 to 6,000 ng/mL. **(B)** A standard curve from **(A)** linear regression shows a working range of the DAS-qELISA from 10 to 1,280 ng/mL for InlG (R^2^ = 0.9913). **(C)** The linear curve of the DAS-qELISA assay for the quantitative detection of serially diluted *L. monocytogenes* from 10^1^–10^9^ CFU/mL. **(D)** The standard curve from (C) shows a working range of the DAS-qELISA assay from 10^2^–10^7^ CFU/mL for *L. monocytogenes*.

For the protein-negative control group, the mean OD_450_ value was 0.179 (Figure 3A). As shown in Figure 3B, linear regression analysis of the InlG protein concentration (ranging from 10 to 1,280 ng/mL) versus the OD_450_ value yielded a regression equation of *y* = 0.0011x + 0.3417, with a high coefficient of determination (R^2^ = 0.9913). Based on this analysis, the limit of detection for InlG protein was determined to be 32 ng/mL.

In the case of the bacterial-negative control group, the mean OD_450_ value was 0.302 (Figure 3C). As shown in Figure 3D, linear regression analysis of the logarithm of bacterial concentration from 10^2^ CFU/mL to 10^7^ CFU/mL versus the assay value produced a regression equation of y = 0.2295x - 0.26, with an R^2^ value of 0.990, indicating a strong correlation. The lowest positive dilution corresponding to a detectable signal was 7875.83 CFU/mL, establishing this as the detection limit for *L. monocytogenes*.

To evaluate the precision and accuracy of the developed ELISA protocol, a series of bacterial concentrations were examined, and the outcomes are delineated in Table 3. The assay exhibited a coefficient of variation (CV) of less than 10% for bacterial concentrations exceeding 10^3^ CFU/mL, which signifies minimal variability, high reproducibility, and robust accuracy. The accuracy of the method across the tested bacterial concentration spectrum ranged from 91.56 to 107.07%, signifying that the empirical values closely corresponded to the expected values, thereby affirming the method’s high degree of accuracy.

**Table 3:** Precision and accuracy of DAS-qELISA for *L. monocytogenes* detection.

Concentration of *L. monocytogenes* ATCC 19111(CFU/mL)	Precision^a^			Accuracy^a^
	Mean	SD	CV^b^ (%)	
10^6^	915,632.59	30,235.11	3.30%	91.56%
10^5^	94,705.91	6,110.60	6.45%	94.71%
10^4^	10,267.67	909.98	8.86%	102.68%
10^3^	1070.67	143.97	13.45%	107.07%

a Each assay was repeated three times, the result of the recovery was the average of three replicates.

b CV was the ratio of the standard deviation to the mean.

**Figure 4:**
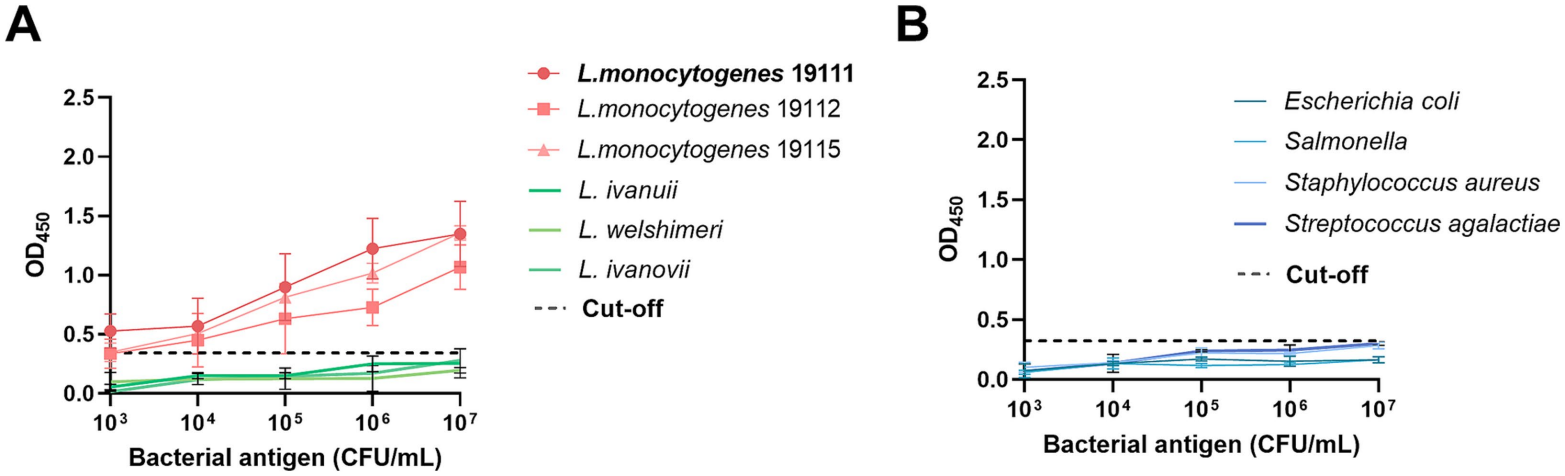
The specificity test determined by DAS-qELISA based on 1D2 mAb and 2H10-HRP mAb. **(A)** Dose–response curve of three *L. monocytogenes* strains with the DAS-qELISA assay. **(B)** Dose–response curve of DAS-qELISA assay for detecting other non-*Listeria* species strains as listed in Table 1.

The specificity of the established ELISA method was further examined utilizing a diverse array of bacterial species. The data revealed that the OD_450_ readings for distinct serotypes of *L. monocytogenes* surpassed the predetermined cut-off value (Figure 4A), indicative of positive detection, whereas the OD_450_ readings for non-*L. monocytogenes* bacterial species remained below the cut-off (Figure 4B), indicative of negative detection. These results confirm that the method exhibits specificity for the detection of *L. monocytogenes*.

### 3.4 Evaluation of sample assay

To evaluate the efficacy of the optimized DAS-qELISA method in detecting *L. monocytogenes* ATCC19111 in food matrices, varying concentrations of the pathogen were used to inoculate sterilized milk and beef samples. Subsequently, these contaminated samples underwent an enrichment phase for a duration ranging from 6 to 24 h. The enriched cultures were subsequently subjected to analysis via the optimized DAS-qELISA protocol, and the findings are detailed in Table 4. Detection of *L. monocytogenes* ATCC19111 was successfully achieved after 24 h of enrichment when the initial contamination level was 10 CFU for both milk and beef samples. Moreover, contamination at a level of 10^3^ CFU was detectable after a 6-h enrichment period in both milk and beef.

**Table 4:** Quantification efficiency of *L. monocytogenes* spiked in milk and beef samples.

Sample	Spiked (CFU/mL)	Enrichment period (CFU/mL)			Control
		6 h	12 h	24 h	
Milk	10^4^	2.34 × 10^5^ ± 3.12 × 10^4^	3.76 × 10^7^ ± 1.82 × 10^6^	1.29 × 10^8^ ± 5.71 × 10^7^	ND
	10^3^	ND	8.22 × 10^6^ ± 8.04 × 10^5^	2.71 × 10^8^ ± 3.59 × 10^7^	
	10^2^	ND	ND	7.72 × 10^7^ ± 2.04 × 10^6^	
	10	ND	ND	2.62 × 10^7^ ± 3.04 × 10^6^	
Beef	10^4^	1.11 × 10^5^ ± 1.05 × 10^4^	6.29 × 10^6^ ± 1.22 × 10^5^	2.12 × 10^8^ ± 5.05 × 10^7^	ND
	10^3^	ND	6.41 × 10^5^ ± 5.97 × 10^4^	7.56 × 10^8^ ± 3.12 × 10^7^	
	10^2^	ND	ND	4.76 × 10^7^ ± 4.53 × 10^6^	
	10	ND	ND	1.76 × 10^7^ ± 6.69 × 10^6^	

The optimized DAS-qELISA method was further applied to a batch of 200 carcass samples procured from slaughterhouses. The outcomes were compared with those obtained from the national standard test method and the PCR technique, and the comparative results are summarized in Table 5. The national standard method identified 16 positive samples, corresponding to a positivity rate of 8%. In the absence of enrichment, the ELISA method yielded no positive results. However, following a 6-h enrichment period, the ELISA method detected 14 positive samples, resulting in a positivity rate of 7%. After a 12-h enrichment period, the ELISA method identified 16 positive samples, yielding a positivity rate of 8%, which aligned with the results obtained from the national standard method.

**Table 5:** Comparison of DAS-qELISA, PCR, and the traditional standard method for detecting *L. monocytogenes* in slaughterhouse environmental samples.

Different assay for detecting *L. monocytogenes*	Enrichment period			
	6 h	12 h	24 h	72 h
Developed sandwich ELISA	8	16	16	16
PCR	20	24	19	22
Plate isolation	-	-	-	16

## 4 Discussion

In recent years, the incidence of *L. monocytogenes* detection in livestock and poultry meats within China has climbed to 7.1%, with even higher prevalence rates observed in abattoir environments, particularly on carcasses, as compared to agricultural settings (24, 25). Furthermore, as a major foodborne pathogen and zoonotic agent, *L. monocytogenes* poses a significant threat to public health due to its ability to contaminate food products and subsequently infect humans. The peril of *L. monocytogenes* infection has intensified concomitantly with evolving dietary preferences, which now include a growing tolerance for raw or undercooked foodstuffs (26). This threat is further exacerbated by the absence of a commercially available vaccine against this pathogen exacerbates the situation. Thus, the rapid and precise detection methods of *L. monocytogenes* in foodstuffs could play a critical role in preventing outbreaks of listeriosis. This is particularly vital given the severe health implications of listeriosis, including high mortality rates and long-term complications in affected individuals. Consequently, the expedient and precise identification of *L. monocytogenes* contamination at the stages of slaughter and distribution is imperative for mitigating the dissemination of this bacterium and the incidence of associated infections. Although conventional techniques such as isolation and biochemical assays are efficacious, they are often protracted and necessitate specialized reagents (27, 28). In contrast, the ELISA presents a more expeditious, sensitive, and technically less demanding approach and have demonstrated broad utility in identifying other foodborne pathogens such as *Salmonella* spp., *E. coli*, *Listeria* and *Campylobacter* (29). For instance, Gu et al. (30) established a nanobody-horseradish peroxidase-based sandwich ELISA method for detecting *Salmonella* in milk and chicken with high specificity and sensitivity, achieving a detection limit of 10 CFU/mL within 8 h of enrichment. Similarly, double-antibody sandwich ELISA and indirectly competitive ELISA assay targeting *E. coli* O157:H7 has been developed, allowing rapid detection in milk (31). Such advancements have significantly reduced detection times compared to traditional culture-based methods, aligning with food industry demands for rapid pathogen identification. By providing a sensitive and reliable tool for the early detection of contamination in food production and distribution systems, this method could help interrupt the transmission of *L. monocytogenes* from contaminated food to humans. Such applications align with broader public health strategies aimed at reducing the burden of foodborne illnesses and controlling zoonotic diseases. Nevertheless, existing ELISA kits designed to detect anti-hemolysin IgG antibodies for *L. monocytogenes* have been associated with deficiencies in accuracy and consistency. Addressing these limitations, our investigation has developed a DAS-qELISA methodology founded on mAbs against InlG, tailored for the specific detection of *L. monocytogenes*.

InlG, a constituent of the internalin protein family secreted by *L. monocytogenes*, is a cell surface-associated protein distinguished by the LPXTG motif (Leu-Pro-X-Thr-Gly, with X denoting any amino acid) (18). Investigations conducted in our laboratory have previously elucidated that the deletion of the inlG gene does not substantially impact bacterial proliferation or environmental resilience; however, it significantly diminishes virulence while concurrently augmenting the adhesion and invasion capabilities of Caco-2 cells, implicating InlG in the pathobiology of *L. monocytogenes* as a cell surface protein (20). Moreover, it has been proposed that InlG is predominantly expressed in *L. monocytogenes* strains of high virulence and in isolates derived from environmental sources (19, 32).

In the present study, our initial objective was to employ InlG-inducible mAbs to differentiate between highly virulent and less virulent *L. monocytogenes* strains. Contrary to expectations, we observed positive detection outcomes utilizing the InlG mAb against *L. monocytogenes* strains that lack the inlG gene within their genomic sequence. We postulate that this unexpected finding may be attributed to the structural attributes of the InlG protein or to cross-reactivity of the antibody with related epitopes. Evidence from the literature suggests that antibodies can occasionally exhibit cross-reactivity with other proteins that possess similar structural motifs or epitopes (33, 34). Additionally, during the processes of protein expression and modification, mutations may arise, potentially uncovering antigenic determinants that mimic those of the InlG protein, thereby eliciting cross-reactivity. Nonetheless, the InlG mAb demonstrated commendable species specificity, resulting in negative detection outcomes for *L. innocua*, other *Listeria* species, and various non-Listeria bacterial genera. Furthermore, the DAS-qELISA assay developed using the InlG mAb exhibited high specificity in the detection of *L. monocytogenes*, without yielding false-positive results from other bacterial genera.

In a prior investigation, a sandwich ELISA was developed wherein a mAb targeting InlG served as the capture antibody, and a pAb directed against *L. monocytogenes* was utilized for detection. Although this methodology yielded a high positive-to-negative (P/N) ratio, the pAb’s recognition of multiple antigenic epitopes resulted in a considerable false-positive rate, thereby compromising assay specificity (21). The objective of the current study was to enhance the specificity of the assay by developing a DAS-qELISA that employs mAbs specific to distinct epitopes for both the capture and detection phases. This refined approach also yielded a P/N ratio exceeding 23, coupled with heightened specificity, prompting the selection of this mAb pair for additional optimization.

The sensitivity and specificity of the DAS-ELISA method are influenced by several variables, with antibody concentration being a critical determinant. An inordinately high concentration of antibodies can lead to an increase in non-specific binding, which subsequently elevates background signal and diminishes specificity (35). Conversely, an insufficient antibody concentration may result in inadequate antigen capture or detection, thereby compromising sensitivity (36). Consequently, the establishment of the optimal working concentration for the antibody pair is of paramount importance. Additionally, the duration of antigen incubation is a factor that affects the efficiency of antigen–antibody binding. Extended incubation periods can augment antigen capture and enhance assay sensitivity; however, excessively prolonged incubation may facilitate non-specific binding, thereby reducing specificity (37). In this study, we systematically refined the assay conditions to enhance sensitivity while maintaining specificity. Subsequent to this optimization, the final assay protocol was assessed for sensitivity, precision, and accuracy. The limit of detection (LOD), indicative of the minimum antigen concentration that yields a positive signal, is a critical metric for evaluating ELISA sensitivity. The LODs for the optimized DAS-qELISA were established using InlG protein and *L. monocytogenes* as the respective antigens. A robust linear correlation was noted between the optical density at 450 nm (OD450) and the concentration of the protein antigen. However, the coefficient of determination (R^2^) for the standard curve was lower when *L. monocytogenes* was employed as the antigen, which may be attributed to greater variability in the bacterial dilution series. Consequently, further assessments of the assay’s accuracy and precision were performed across a range of bacterial concentrations. The coefficient of variation (CV) was found to be less than 10% for bacterial concentrations exceeding 10^3^ CFU/mL, indicative of high precision. The accuracy of the assay spanned from 90 to 110%, conforming to the standards expected of commercial ELISA kits (38, 39). The established DAS-qELISA method with the detection limit of 7362.07 CFU/mL retains significant practical utility. An indirect ELISA previously developed for the detection of *L. monocytogenes* InlA and InlB proteins has demonstrated adequate specificity and sensitivity, yet the determination of its detection limit has not been undertaken and should be the subject of future research (17). An alternative sandwich ELISA utilizing single-chain variable fragments (scFv) and mAbs for the detection of *L. monocytogenes* yielded a detection limit of 10^6.5^ CFU/mL (40), which is higher than that achieved by the method developed herein. These findings suggest that the DAS-qELISA method based on InlG mAbs surpasses existing methodologies in terms of detection limit and is capable of quantitatively assessing *L. monocytogenes* in various samples. Additionally, its accuracy and precision are aligned with the benchmarks established for commercial ELISA kits.

Currently, the conventional method for detecting *L. monocytogenes* in meat within China is culture-based identification, a process that entails over 12 h of enrichment and subsequent plate isolation, leading to an overall detection period of approximately 3–5 days. Despite its high accuracy, this approach is both time-consuming and labor-intensive. In the present study, we conducted a comparative evaluation of the time required for detection and the outcomes derived from the application of the traditional culture method, polymerase chain reaction (PCR), and the newly developed DAS-qELISA assay. Our findings revealed that the DAS-qELISA assay was unable to detect *L. monocytogenes* in unenriched samples; however, following a 12-h enrichment period, the detection rate achieved parity with the traditional culture method, thereby markedly reducing the time to detection. Although the PCR technique permits direct detection of *L. monocytogenes* from samples without enrichment, it is encumbered by limitations such as reduced accuracy and an elevated false-positive rate. In conclusion, this research successfully established a DAS-qELISA method predicated on the InlG protein of *L. monocytogenes* for the detection of this pathogen in food products. The developed method provides a low detection limit, high specificity, and robust accuracy and precision, while concurrently diminishing the detection time and cost. Its utility in the production process has the potential to contribute to the prevention of *L. monocytogenes* transmission via foodstuffs.

## Data Availability

The original contributions presented in the study are included in the article/supplementary material, further inquiries can be directed to the corresponding authors.
